# On the homogeneity and heterogeneity of cortical thickness profiles in Homo sapiens sapiens

**DOI:** 10.1038/s41598-017-17154-y

**Published:** 2017-12-20

**Authors:** Jan Willem Koten, André Schüppen, Maria Morozova, Agnes Lehofer, Karl Koschutnig, Guilherme Wood

**Affiliations:** 1 Department of Psychology, Karl-Franzens-University of Graz, Graz, 8010 Austria; 2 Biotechmed Graz, Graz, 8010 Austria; 3 Brain Imaging Facility of the Interdisciplinary Centre for Clinical Research of the University Hospital Rheinisch-Westfälische Technische Hochschule (RWTH), Aachen, 52074 Germany

## Abstract

Cortical thickness has been investigated since the beginning of the 20th century, but we do not know how similar the cortical thickness profiles among humans are. In this study, the local similarity of cortical thickness profiles was investigated using sliding window methods. Here, we show that approximately 5% of the cortical thickness profiles are similarly expressed among humans while 45% of the cortical thickness profiles show a high level of heterogeneity. Therefore, heterogeneity is the rule, not the exception. Cortical thickness profiles of somatosensory homunculi and the anterior insula are consistent among humans, while the cortical thickness profiles of the motor homunculus are more variable. Cortical thickness profiles of homunculi that code for muscle position and skin stimulation are highly similar among humans despite large differences in sex, education, and age. This finding suggests that the structure of these cortices remains well preserved over a lifetime. Our observations possibly relativize opinions on cortical plasticity.

## Introduction

The universal expression of phenotypes in species suggests the innateness within that species. For example, most humans walk on two feet while this is rarely the case in other animals. Gross brain anatomical structures are also innate. For instance, most humans appear to possess two halves of the brain. Are subtler aspects of brain anatomy universally expressed as well? Currently, several structural imaging methods exist that may shed light on the latter question. The white matter in the nervous system is often studied with diffusion tensor imaging, while the gray matter is often studied on the basis of T1-weighted imaging procedures^1^. It is possible to analyze gyrification patterns, cortical folding depth, and cortical thickness from T1 images^1–4^. Cortical thickness has been studied with a rich repertoire of statistical techniques including variance analysis, principal component analysis, structural equation modeling, and classification methods^4–7^. It is possible to relate individual differences in cortical thickness to psychiatric^6^ and neurological^7^ diseases as well as to age^8,9^. Finally, cortical thickness is partly under genetic^6,8,10^ and evolutionary control^2,9^.

Although cortical thickness has been investigated since the beginning of the 20th century^11–13^ we still do no not know how similar local cortical thickness profiles are expressed in humans. Perhaps this question never arose because “*it is currently accepted that cortical maps are dynamic constructs that are remodeled in detail by behaviorally important experiences throughout life*”^14^. Alternatively, one might argue that it is difficult to study local similarity of cortical thickness profiles (LSCTP) due to the inherent mismatch in brain alignment among humans^1–3^.

**Figure 1:**
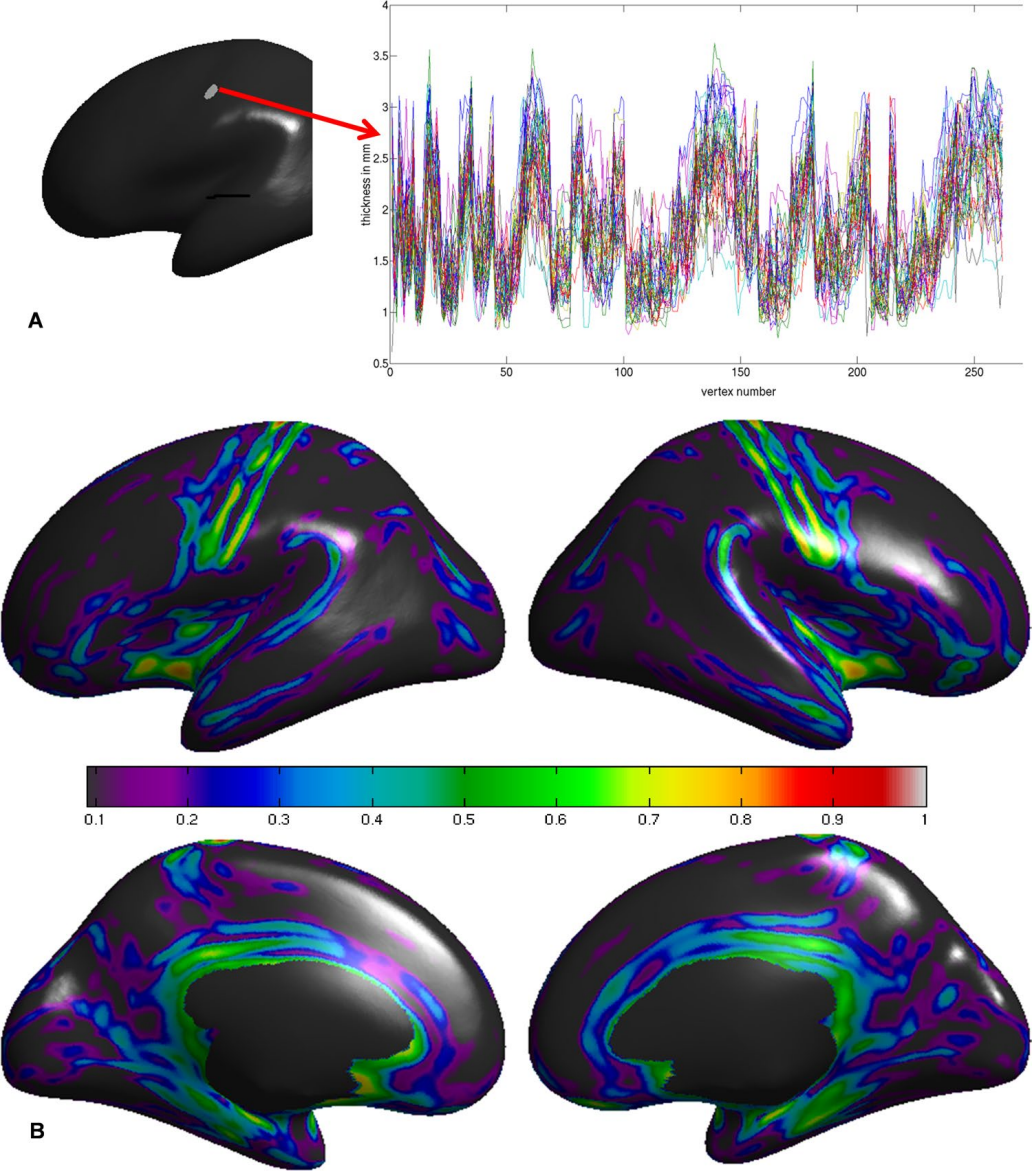
(**A**) Local similarity of cortical thickness profiles extracted from a region of interest in the central sulcus (window diameter = 8 mm). Each line depicts the thickness profile of one of the 42 individuals investigated. In this region of interest, mean correlation among individuals was 0.78. (**B**) Whole brain depiction of local similarity of cortical thickness (window diameter = 8 mm). The colors of the color bar represent the height of the average correlation among individuals. Maps were thresholded at p < 10^−6^.

Here, we introduce a sliding window-based analysis method that accounts for the spatial context of cortical thickness in a particular vertex. We studied LSCTP by drawing a circumference around each vertex and extracting the cortical thickness data of all the other vertices contained in this window (Fig. 1). The cortical thickness within this window was extracted for all 42 individuals (half of them female) in this study and was correlated, leading to (42*41)/2 = 861 correlations per vertex. Subsequently, correlations were averaged vertex-wise and tested for their statistical significance using Monte Carlo simulations. We minimized brain alignment mismatch using advanced methods. Finally, we shed light on the functional and histological meaning of our results in a comparative map analysis.

## Results

### Local similarity in cortical thickness profiles

Quantitative and qualitative analyses suggested that maps obtained with sliding window sizes with a diameter between 4 and 8 mm show sufficient anatomical differentiation (Supplementary Fig. S1; Supplementary Figs S3–S6). For a global brain analysis, we opted for a window size of 8 mm that exhibits higher power to detect LSCTPs (Supplementary Figs S3–S6; Supplementary Tables S1–S3). Using Cohen’s correlation criteria, we concluded that approximately 5% of the cortex surface shows high LSCTPs (average *r* > 0.5), while 45% of the cortex shows low LSCTPs (average r < 0.1)^15,16^. Monte Carlo simulations revealed that even low LSCTP values cannot be created by chance (Supplementary Tables S1–S3). This finding suggests that almost the entire cortex exhibits some degree of LSCTP despite inherent mismatches in cortical folding patterns (Supplementary Figs S3–S6; Supplementary Tables S1–S3). The latter is particularly true when larger sliding windows are used. The maps depicted in Fig. 1 reveal that humans exhibit high LSCTP in sensorimotor, anterior insular, and limbic cortices. The latter includes inferior frontal and parahippocampal structures. The highest LSCTP was observed in and around the central sulcus.

### Local similarity in cortical thickness profiles in a functional context

We investigated the overlap in the LSCTP maps with seven functional network-maps from the Freesurfer reference atlas that were obtained through a clustering of resting state data^17^. First, LSCTP maps were created with window diameters between 4 and 32 mm. Next, we estimated the average LSCTP for each functional network and window size.

**Figure 2:**
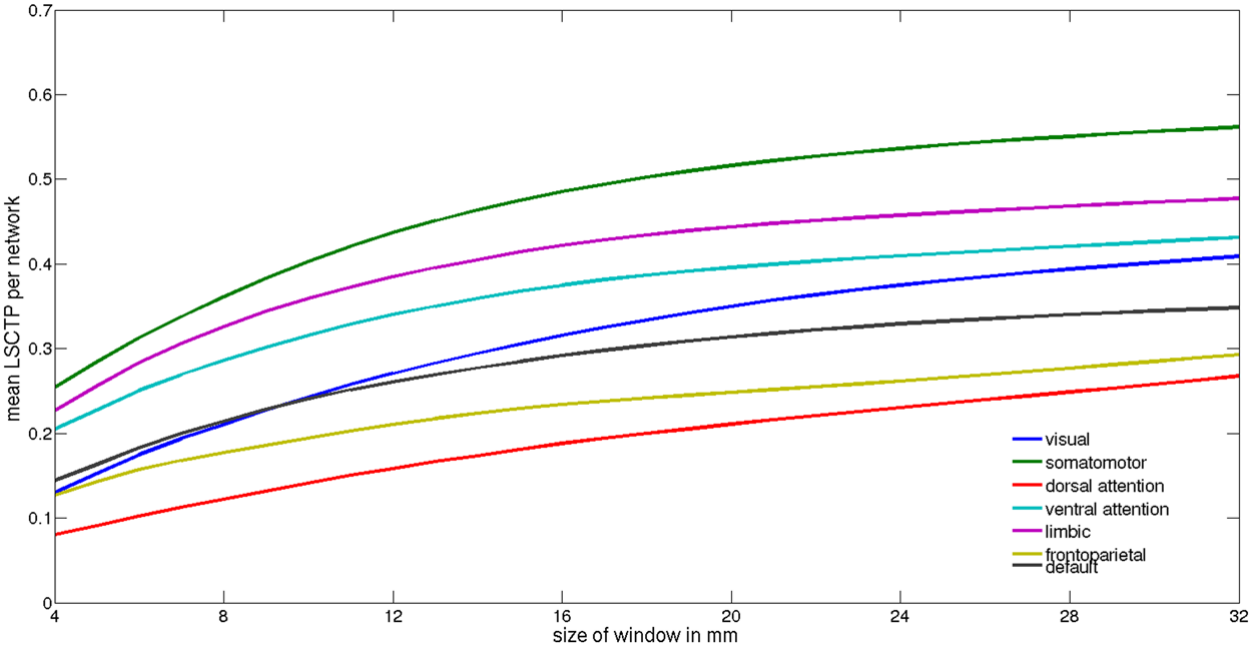
Average local similarity of cortical thickness profiles per functional network depicted as a function of window diameter from 4–32 mm.

Figure 2 reveals that two observations can be made. (i) LSCTP values increase with growing window size and differ across functional networks. These effects cannot be explained by chance alone (Supplementary Table S4). (ii) With one exception, the rank of the functional network does not depend on the window size chosen. The highest LSCTP is observed in evolutionarily old somatomotor and limbic systems^2^. By contrast, the lowest similarity is found in two parietal networks labeled the fronto-parietal and dorsal attention networks^17^. Finally, visual, ventral attention, and default networks are characterized by medium similarity in cortical thickness expression^17^.

### Local similarity in cortical thickness profiles in a histological context

We also investigated the overlap of the LSCTP maps with histological maps^18^. We focused on structures in and around the central sulcus because BA4, in particular, can be aligned with higher precision when spherical alignment procedures are used^18^. We chose a window of 4 mm because it led to maps of higher spatial resolution (Supplementary Figs S3–S6). BA4 is believed to be equivalent to the motor homunculus and is often subdivided into the BA4a and BA4p3 areas, while the primary sensory homunculus is believed to be located in BA1-BA3^18^.

**Figure 3:**
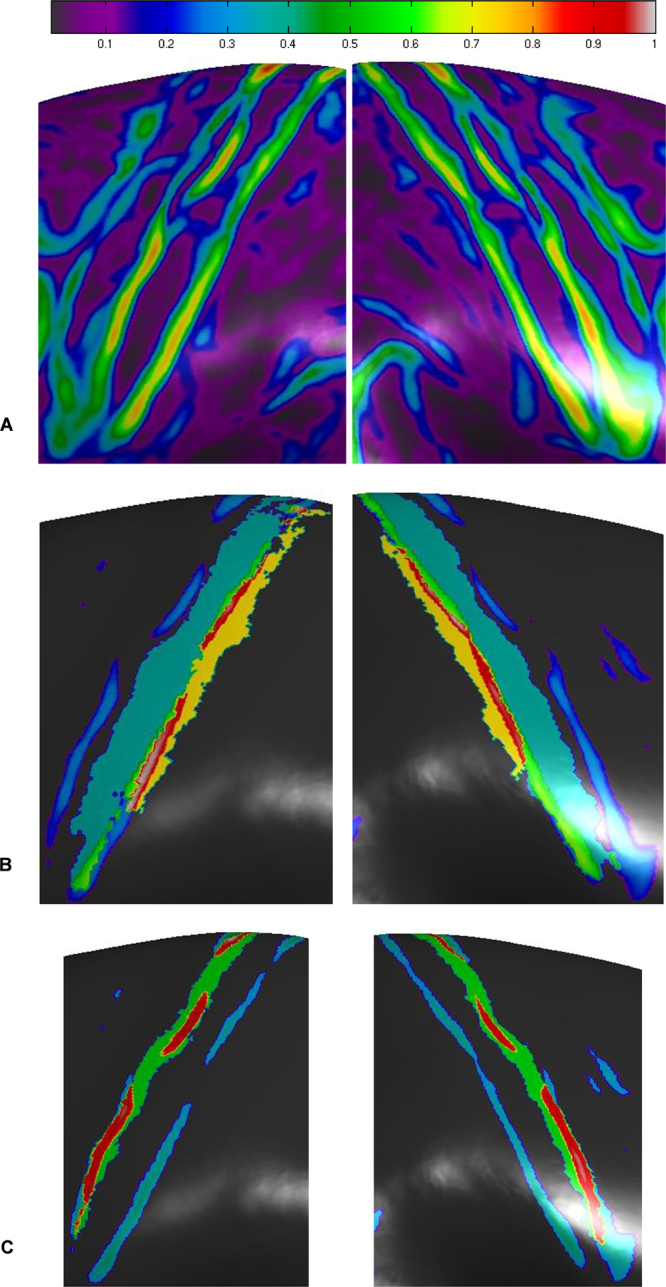
Local similarity of cortical thickness profile maps as obtained with a sliding window diameter of 4 mm are depicted for structures in and around the central sulcus. (**A**) Non-thresholded maps. (**B**) The overlap of local cortical thickness profiles (threshold > 0.5) with BA3b is shown in light green. The overlap with BA1 is shown in red. Non-overlapping parts of BA3a and BA1 are depicted in blue-green and orange, respectively. (**C**) The overlap of local cortical thickness profiles (threshold > 0.5) with BA3a is shown in red. Non-overlapping parts of BA3a are depicted in green.

Figure 3 reveals that the LSCTP in structures around the central sulcus is symmetrically expressed. Together with histological brain masks^18^, we depicted high LSCTPs > 0.5 that, according to Cohen, can be classified as high^15,16^. High LSCTPs are observed in BA3a and in structures bordering the BA3b/BA1 region. This finding demonstrates that parts of the sensory homunculus organization are highly similar across humans. By contrast, we found only a small number of LSCTPs > 0.5 in BA 4 (Supplementary Fig. S8). The lack of high LSCTPs in the motor homunculus is remarkable because it is spatially adjacent to BA3. Hence, structural differences found in the neighboring regions of cortices BA 4 and BA 3 suggest that high LSCTPs observed in BA 3 are not artifacts.

## Discussion

*The highest LSCTP was observed in evolutionarily old limbic and somatomotor systems* ^2,19^. Classic and modern research has demonstrated that plastic changes in the form and function of the homunculi occur when an organism is subjected to experimental manipulation or suffers from disease^14,20–31^. We do not dispute the existence of plasticity in the somatosensory homunculus. However, our experiment suggests that plastic changes in the cortical thickness profiles of BA1/BA3 are less likely when individuals are exposed to normal environmental differences. Altogether, differences in age, sex, and education level in the normal range do not seem to affect the local similarity of cortical thickness profiles of BA3/BA1. We speculate that plastic changes in the cortical thickness profiles of the homunculi mainly occur when the organism under study is the subject of extreme experimental manipulation or severe disease. We conclude that humans have similar cortical thickness profiles in areas that code for muscle position (BA3a) and skin stimulation (BA3b BA1)^20^. The high similarity of cortical thickness profiles suggests that these areas are innate in nature. Genetic analysis of the central sulcus depth shows that the shape of the central sulcus is indeed driven by genetic factors^4^. Recent research executed on a microscopic level suggests that neuronal representations are stabilized by behavioral relevance. In this context, the degree of long-term tuning stability and perturbation resistance is related to the functional role of the respective neurons^32^. *LSCTP maps may provide new insights on neuroanatomy, but the method also shows serious limitations that are discussed below*. We have shown that the cortical thickness profiles of BA3 and BA1 are highly similar among humans. Thus, this aspect of the shape of the cortex does not seem to be plastic. The generalizability of our results is limited by the nature of the method. The graph presented in Fig. 1 reveals that some individual differences in cortical thickness exist. It is possible that the environment shapes cortical thickness at the vertex level while it has little effect on the shape of the cortical profile. Furthermore, we cannot exclude the possibility that plastic changes occur on a microscopic or functional level^14^. Accordingly, it would be interesting to investigate if the thickness profiles of histological layers are similarly expressed among humans. Moreover, some cortical structures could not be included in our analysis since one cannot compute LSCTP of structures with complex folding patterns such as the hippocampus. Finally, the spatial resolution of our experiments is only one millimeter. This is not much given that some cortices are indeed very thin^2^. Hence, our method may benefit substantially from ultra-high field imaging, which allows for capturing images of higher spatial resolution^1^.

While insular and somatosensory cortices display a high amount of LSCTP, this is less the case for other cortices. Dissimilarities in cortical thickness profiles may be caused by individual differences in genetic makeup^6,8,10^ or differences in life experiences that may occur with plastic changes. However, dissimilarities among individuals may also arise from alignment problems. Surface-based alignment procedures belong to the most sophisticated alignment methods currently available and usually outperform 3D alignment methods^1–3,18^. Nevertheless, the precision of surface-based alignment is limited. While large sulci and gyri are indeed mapped accurately, this is less the case for smaller gyri and sulci that are characterized by large individual differences^1^. Given these problems, it is quite likely that LSCTP has been underestimated. In Fig. 2 and Supplementary Figs. S3–S6, we show that LSCTP increases when larger sliding windows are used. Tackling alignment problems through larger sliding windows cannot be the final answer to the alignment problem because it destroys the effective spatial resolution of the map. Hopefully, better alignment techniques will be developed^1^. As has been noted, (f)MRI group studies are problematic^1–3^. The basic assumption is that human brains are sufficiently similar to justify averaging procedures. On the basis of our results, we are somewhat skeptical. In fact, the cortical profiles of human brains that have been treated with sophisticated alignment procedures are mainly dissimilar, and being similar is the exception.

## Methods

### Sample composition

All individuals provided informed consent. The research was executed according to the guidelines of the Declaration of Helsinki. The study was approved by the Ethics committee of the University of Graz under GZ 39/31/63 ex 2011/12. The study consisted of 21 males and 21 females with an age range of 21–46 years. The educational level of each participant is representative of the Austrian population. Thirteen females had a lower to medium education level (mean rounded age = 32 years), and eight females had a higher education level (mean rounded age = 31 years). Thirteen males had a lower to medium education level (mean rounded age = 31 years), and eight males had a higher education level (mean rounded age = 32 years).

### MRI scanner protocol

MRI scans were performed on a 3 T Siemens Magnetom Skyra (Siemens Medical Systems, Erlangen, Germany). We used a 3D-MPRAGE sequence (176 slices per slab, FOV = 256 mm, TR = 2530 ms, TE = 2.07 ms, TI = 900 ms, Flip angle = 9°, voxel size = 1 mm isotropic) with a 32-channel head coil.

### Segmentation

All scans were processed using the standard recon all pipeline of Freesurfer by KK^33^. The resulting segmentations were visually inspected, and hand corrections were made when needed by AL and MM. Both coauthors were instructed on the art of segmentation by the first author. The hand-corrected segmentations were inspected by the first author and by KK. Next, the segmented maps were reprocessed in Freesurfer by KK such that images with correct cortical thickness were obtained. The cortical thickness data were brought into the FS_average space using spherical alignment procedures, but we did not apply spatial smoothing because this may level out the data profiles and reduce artificially the spatial resolution of the images.

### Sliding window analysis

Determining the size of the sliding window is not trivial. We chose to find the optimal window size empirically. For this reason, we searched within the limits of a larger window space ranging from 2 to 32 mm. The lower limit of this space was given by the number of vertices that might estimate meaningful correlations, while the upper limit was chosen such that the diameter of the window exceeded the sulcal depth of the central sulcus. The optimal window size was determined in a two-stage approach. In the first stage, we created LSCTP maps. In the second stage, we investigated the local variability in LSCTPs. We defined subsets of LSCTPs by drawing a circumference around a specific mesh element that represented a surface along the border of the white matter and the cortex (see Fig. 1 for an example). Next, cortical thickness measurements within this window were extracted for all 42 individuals in the study and were correlated, leading to (42*41)/2 = 861 correlations. Subsequently, the 861 correlations were averaged using fisher’s z’ transformation. We extracted the data using the Matlab/Surfstat function “SurfStatRoi”^5^ that was embedded in our in-house Matlab code and used the standard sphere as the target mesh. The latter is used for co-registration purposes. We estimated 30 LSCTP maps starting with a window size of 2 mm and ending with a window size of 32 mm. We depicted LSCTP maps of 4, 8, 16, and 32 mm in Supplementary Figs S3–S6. We decided to exclude vertices from the analysis when at least one individual exhibited a cortical thickness of zero within the sliding window. Thus, the size of the black masks that do not contain information and that were visible on the medial side of the mesh increased with increasing diameter of the sliding window. The number of vertices that were subject to analysis is shown in Supplementary Tables S2–S3.

The local variability in LSCTP values provides information on the amount of anatomical differentiation. For this analysis, the variability in LSCTPs within the sliding window was computed for the 8, 16, and 32 mm window diameter. That is, the 30 LSCTP maps that were obtained in the previous steps were again analyzed with a sliding window method. The standard deviation in the LSCTP correlations within the sliding window was employed as a measurement of variability. Next, we averaged the standard deviation in the LSCTP maps per window diameter. The resulting graph depicted in Supplementary Fig. S1 suggests that the highest standard deviations were obtained with primary window sizes that ranged from 4 mm to 8 mm. Visual inspection of the data indeed suggests that maps with window sizes smaller than 4 mm and larger than 8 mm contain less detailed anatomical information. Given these results, we decided to use a window of 8 mm for a global LSCTP map and a window of 4 mm for an LSCTP map that focused on the central sulcus.

### Spatial smoothness estimation and spatial smoothing

As described above, we did not apply spatial smoothing to the original thickness data because this may level out thickness profiles and destroy the spatial resolution of images. However, even unsmoothed data exhibit natural spatial smoothness that must be accounted for when Monte Carlo simulations are performed. Estimating the spatial smoothness of the data in normal 3D image space is fairly easy^34^, as it is relatively easy to smooth out 3D data^34^. However, for 2D meshes, estimating the spatial smoothness is less straightforward due to the spatial organization of the data. Data on 2D meshes is organized in a “pinwheel” fashion, where every vertex of the “pinwheel” may have a different size. Spatial smoothness was estimated using following formula^34^:

1 $${\rm{F}}{\rm{W}}{\rm{H}}{\rm{M}}\,{\rm{s}}{\rm{u}}{\rm{r}}{\rm{f}}={\rm{d}}{\rm{v}}\ast \surd (-2\ast \,{\rm{l}}{\rm{n}}\,2/{\rm{l}}{\rm{n}}(1-{\rm{v}}{\rm{a}}{\rm{r}}({\rm{d}}{\rm{s}})/2\ast {\rm{v}}{\rm{a}}{\rm{r}}({\rm{s}})))$$

where dv is the average inter-neighbor distance, var(ds) is the variance seen among inter-neighbor differences, and var(s) is the overall variance in the values at each vertex. In this study, instead of heat kernel or Gaussian smoothing, a simple nearest neighbor smoothing was used^34^. Nearest neighbor smoothing mimics the effects of conventional Gaussian smoothing^34^. Nearest neighbor smoothing is estimated by averaging the thickness data from neighbor vertices^34^. The product of this averaging procedure is again averaged iteratively until the target smoothness is achieved. Nearest neighbor smoothing was implemented using the Freesurfer/Matlab command fs_smooth^34^.

### Monte Carlo simulation

**Figure 4:**
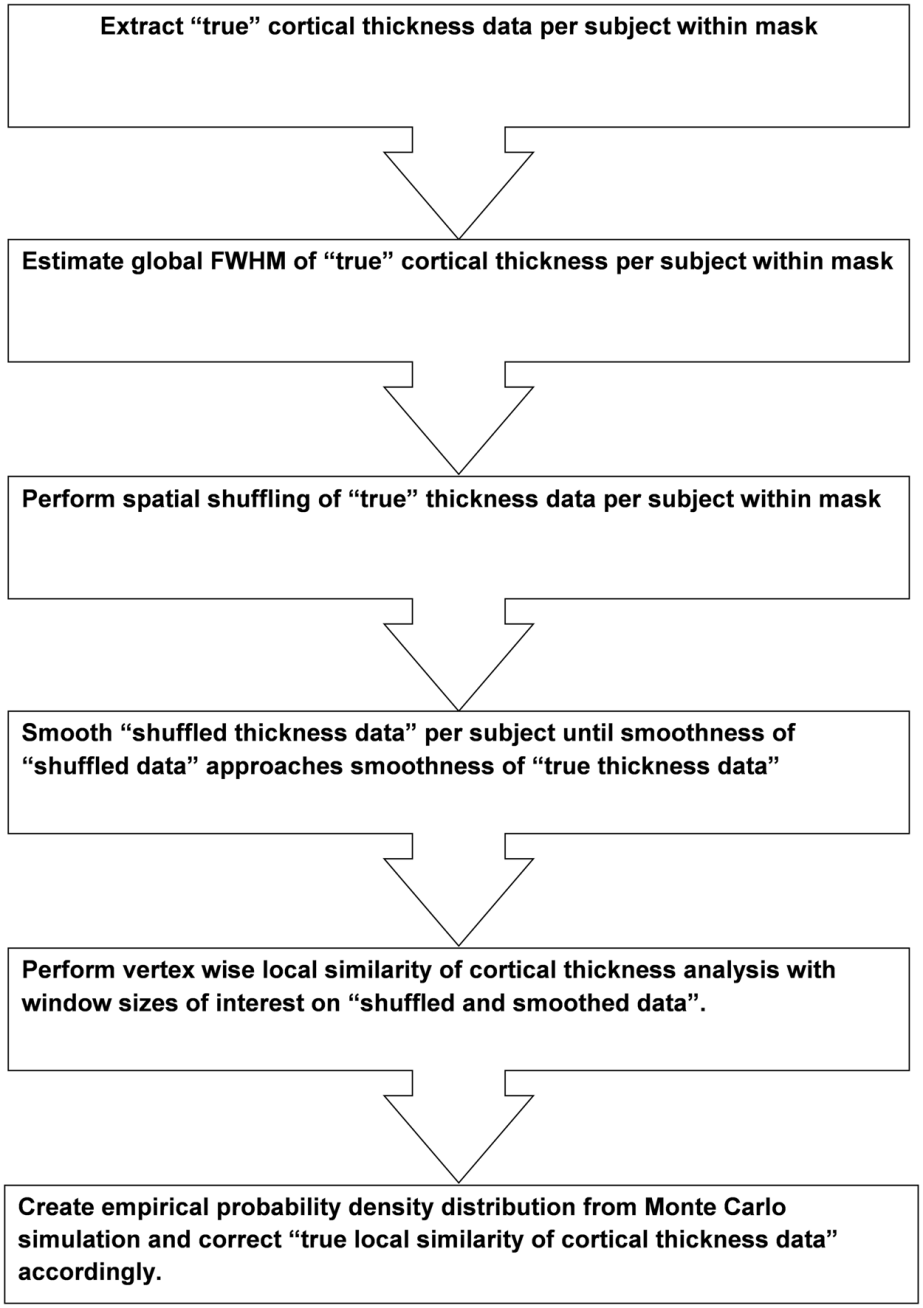
Steps of simulation pipeline used for statistical thresholding purposes.

We adapted several methods used by others^5,33,34^ and depict the novel pipeline in Fig. 4. We placed a threshold on the LSCTP maps using the expected likelihood of finding an LSCTP value for a given window diameter by chance. In short, the global smoothness of the observed data—referred to here as true data—is estimated per subject for all vertices within the gray matter mask^34^ (see the next section for a justification of the mesh reference space). Subsequently, the cortical thickness data is spatially shuffled per subject within the whole surface of the gray matter mask. The global smoothness of the shuffled thickness data is again estimated per subject and compared with the spatial smoothness of the individual’s true data. In other words, we smoothed the shuffled data for a larger iteration space and observed at which iteration step the smoothness of shuffled data approached the smoothness of the true data. The spatial smoothness of the true and shuffled data was estimated with Formula (1). Finally, we extracted the shuffled and smoothed thickness data and estimated the randomized LSCTP maps using the window sizes of 4, 8, 16, and 32 mm. This was done for all vertices within the gray matter mask and repeated 20 times leading to at least 4142820 simulations. From the 20 shuffled maps, the empirical probability distributions of LSCTP values were created. We report the critical LSCTP thresholds for the distinct windows in Supplementary Table S2. We have also masked maps that were created with smaller sliding window sizes with the mask belonging to a sliding window size with a diameter of 32 mm. This operation guarantees compatibility for the distinct window sizes. In addition, we report critical LSCTP thresholds for simulations without spatial smoothing of shuffled data in Supplementary Table S3. A comparison between smoothed and non-smoothed Monte Carlo simulations demonstrates that critical thresholds react sensitively to the number of smoothing iterations performed. For instance, the critical LSCTP value (p < 10^−6^) for a window size of 8 mm is 0.09 for smoothed data but 0.01 for unsmoothed data. This confirms our previous observations that performing the correct number of iterations is crucial in Monte Carlo simulations^33^.

In the main text, we report that the average LSCTP values obtained for the seven functional networks increase when larger gliding windows are used (Fig. 2). As described above, 20 Monte Carlo maps were available for the four window sizes in our study. LSCTP values were extracted per functional mask from the Monte Carlo maps present and were subsequently averaged leading to 20*7 mean values per window size. Subsequently, the 20 averages were averaged again per mask per window. The 7 grand mean values per sliding window size represent the random correlation strength per functional mask, which is without exception extraordinarily low (Table S4).

### Reference Space

In this study, a Monte Carlo simulation was executed using the standard “sphere.reg” files that are used for inter-subject registrations^33,34^. This is a good solution for the following reason: Inter neighbor geometry is more or less constant on any coordinate of the sphere. What follows is that the sliding window contains exactly the same number of vertices on any coordinate of the mesh. Thus, the degrees of freedom used in the statistic are fair for every window. However, this approach can be criticized as well. From the Formula (1) given above, it follows that spatial smoothness depends on the correct estimation of the inter-neighbor distance dv. It has been suggested that only the so-called “smoothwm” meshes obtained in the native space show the correct average inter-neighbor distance of dv = 0.8 mm, which can be used to correctly estimate spatial smoothness^34^. The inter-neighbor distance of the target sphere.reg file is dv = 0.9438, while the smoothwm mesh in FS_average space exhibits dv = 0.6963. A rough estimate of the average spatial smoothness of true data lead to an FWHM = 4.94 and FWHM = 3.64 for the Fs_average sphere.reg and the Fs_average smoothwm targets, respectively. Thus, spatial smoothness estimates depend on the target mesh due to the nature of dv. The latter can be taken from Supplementary Table S1 where the smoothness of the observed data are estimated from Formula (1) and is reported per subject. The exact nature of dv is particularly critical when the number of smoothing iterations that need to be performed on shuffled data is predicted on the basis of a function that is fed with observed FWHM of the true data. According to Hagler^34^:

2 $${\rm{F}}{\rm{W}}{\rm{H}}{\rm{M}}\approx 1.25\ast \surd {\rm{N}}$$

where N represents the number of the nearest neighbor-smoothing iterations needed to obtain a target FWHM. In other words, the number of smoothing iterations critically depends on the true estimation of dv that in turn depends on the mesh in use. We have avoided these problems using the procedure described in the Monte Carlo section of this paper. We smoothed the shuffled data for a larger iteration space and observed at which iteration step the smoothness of shuffled data approached the smoothness of the observed data. We performed this operation using the Fs_average sphere.reg and the Fs_average smoothwm target mesh with dv = 0.9438 and dv = 0.6963, respectively. As can be seen from equation (1), dv is only a constant and not necessary when the formula is used for comparison purposes. As noted, the exact estimate of the spatial smoothness is not important for Monte Carlo simulations when the spatial smoothness of shuffled data approaches the spatial smoothness of true data. As expected, the average number of smoothing iterations needed to obtain spatial smoothness was identical for the different target meshes due to the nature of our algorithm. The average number of iterations needed for the FS_average sphere.reg file with dv = 0.9438 and FS_average smoothwm with dv = 0.6963 was 12.07. This finding proves that our method delivers the right number of smoothing steps regardless of the mesh in use. In some cases, small differences in the number of iteration steps were found between the two target spheres depending on the individual of interest. These small differences are due to rounding error. We report these differences per subject in Supplementary Table 1.

### Data availability statement

The datasets generated and/or analyzed in the current study are available from the corresponding author upon request.

## Electronic Supplementary material

Supplementary information
