# Supplementary material for: On the homogeneity and heterogeneity of cortical thickness profiles in Homo sapiens sapiens
Source: Sci Rep. 2017 Dec 20;7:17937. doi: 10.1038/s41598-017-17154-y (PMC5738339; doi:10.1038/s41598-017-17154-y)
Supplement: Supplementary file 1 — Supplementary information [file 41598_2017_17154_MOESM1_ESM.pdf]

Supplementary Information

On the homogeneity and heterogeneity of cortical thickness profiles in Homo sapiens sapiens.

J.W. Koten jr.[1,3*], A. Schüppen[3], M. Morozova[1], A. Lehofer[1], K. Koschutnig[1,2], G. Wood[1]

1) Department of Psychology, Karl-Franzens-University of Graz, Graz, 8010, Austria
2) Biotechmed, Graz, 8010, Austria
3) Brain Imaging Facility of the Interdisciplinary Centre for Clinical Research of the University Hospital Rheinisch-Westfälische Technische Hochschule (RWTH), Aachen, 52074, Germany

Supplementary Information 1: Local variability of LSCTP

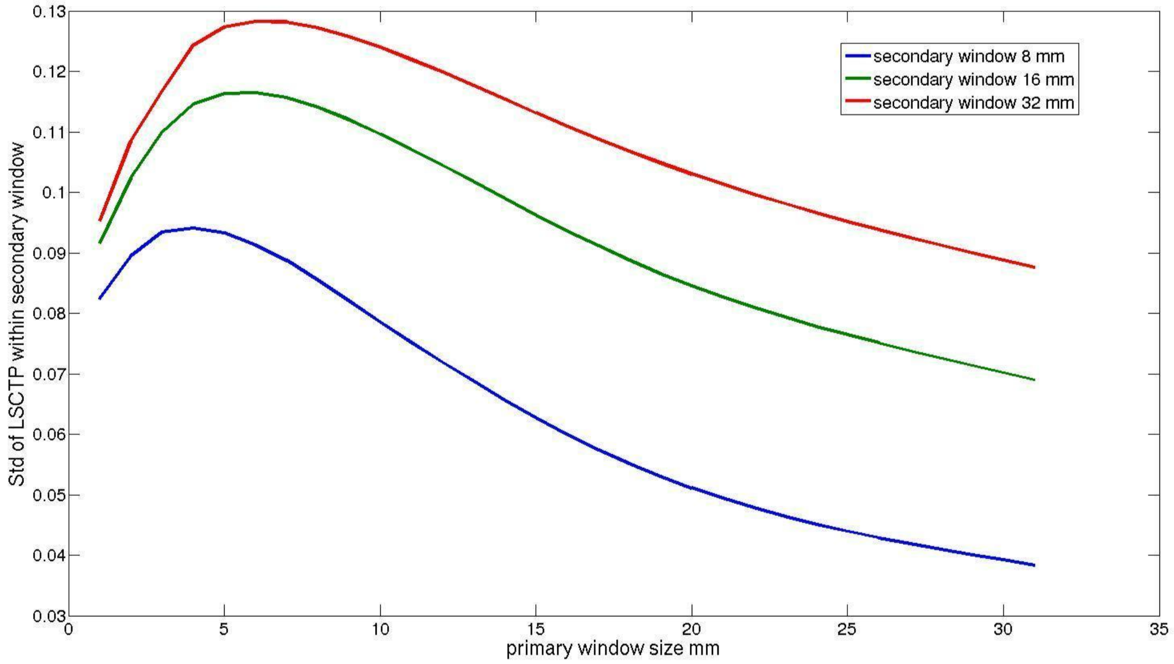

Supplementary Figure S1: Depicts the relation between local standard deviation of local similarity of cortical thickness profiles values (vertical axis) and the window diameter employed to estimate them (horizontal axis). Local similarity of cortical thickness profiles maps that exhibit a high local variability are more informative compared to maps that show low local variability. Results suggest that highest standard deviations were found for primary windows within the 4 to 8 mm range.

Supplementary Information 2: Thickness maps, LSCTP maps, and Local deviation of cortical thickness maps

Here we present the average cortical thickness of our sample (Supplementary Figure S2) that is well in line with observations made by others[1]. Next, we present detailed LSCTP maps as obtained for the various sliding window sizes (Supplementary Figure S3-S6). We have corrected the maps for multiple testing using Monte-Carlo methods. The correction table that resulted from the Monte Carlo analysis is given in Supplementary Information S3. We also present local standard deviations of cortical thickness (LDCT) maps (Supplementary Figure S7). These maps were created only for the 8 mm window that was the primary window of interest. In short, we extracted all thickness data within the window of interest for all 42 subjects. Next we estimated within window standard deviation from the number of subjects*number of vertex thickness data. One might expect that high LSCTP values only occur in regions were sufficient local variance exist. The LDCT maps suggest that high LSCTP values indeed occur in regions that exhibit higher standard deviations. The latter observation was somewhat less the case for the somatosensory system. In particular, the more dorsal aspects of the lateral frontal parietal system were characterized by low LDCT values. This is well in line with the low LSCTP values for frontal parietal systems reported in figure 2 of the main text. In addition regions of higher LDCT and LSCTP can co-occur with higher cortical thickness.

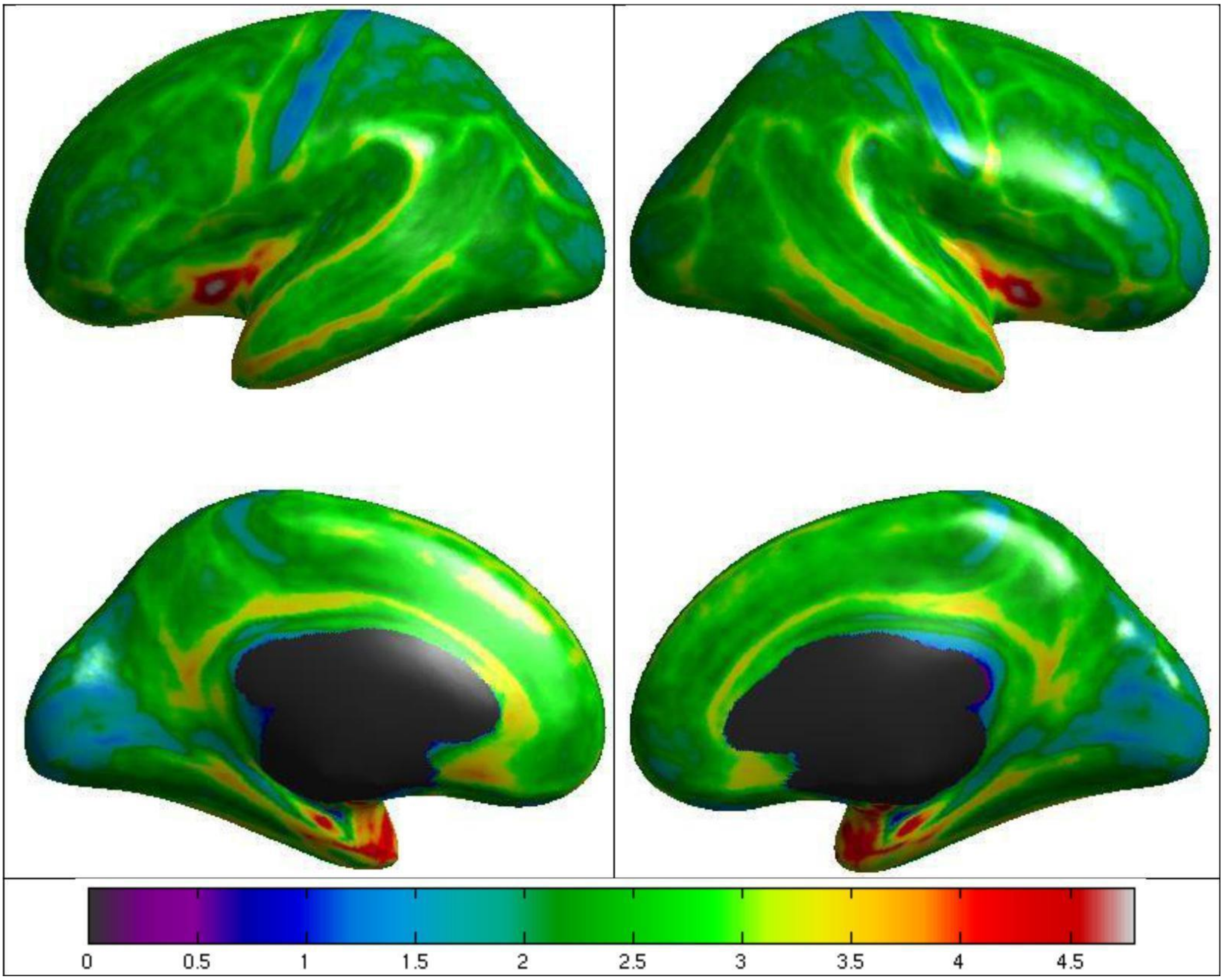

Supplementary Figure S2: Cortical thickness maps averaged from n = 42 individuals. Color code expressed in mm.

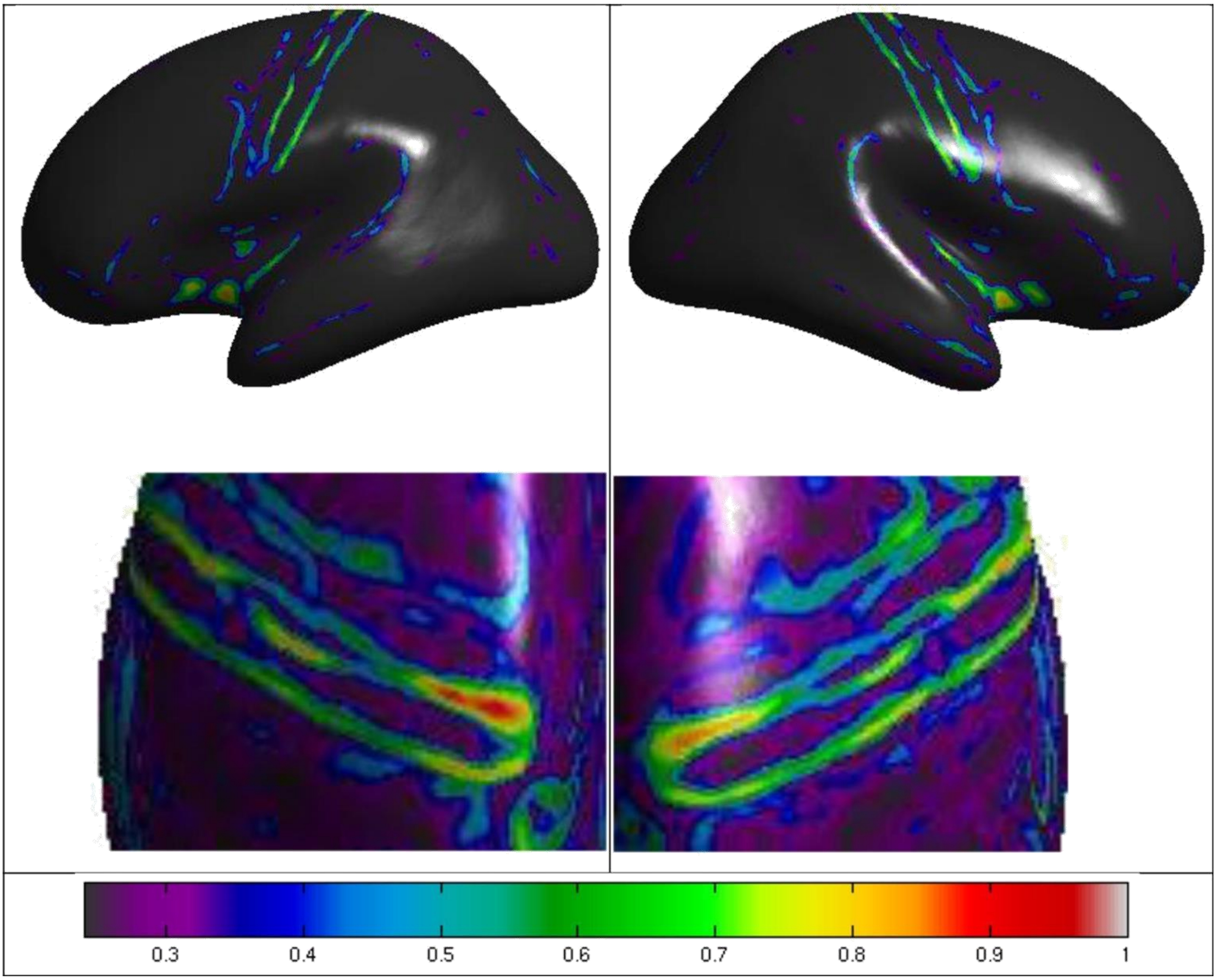

Supplementary Figure S3:

TOP Local similarity of cortical thickness profiles maps obtained with a sliding window with a diameter size of 4 mm covering on average 64.4 vertices. Maps were thresholded at $p < 10^{-6}$. Color code expresses average correlation.

BOTTOM: Unthresholded local similarity of cortical thickness profiles map. Detail depicting a bird eyes view of the central sulcus.

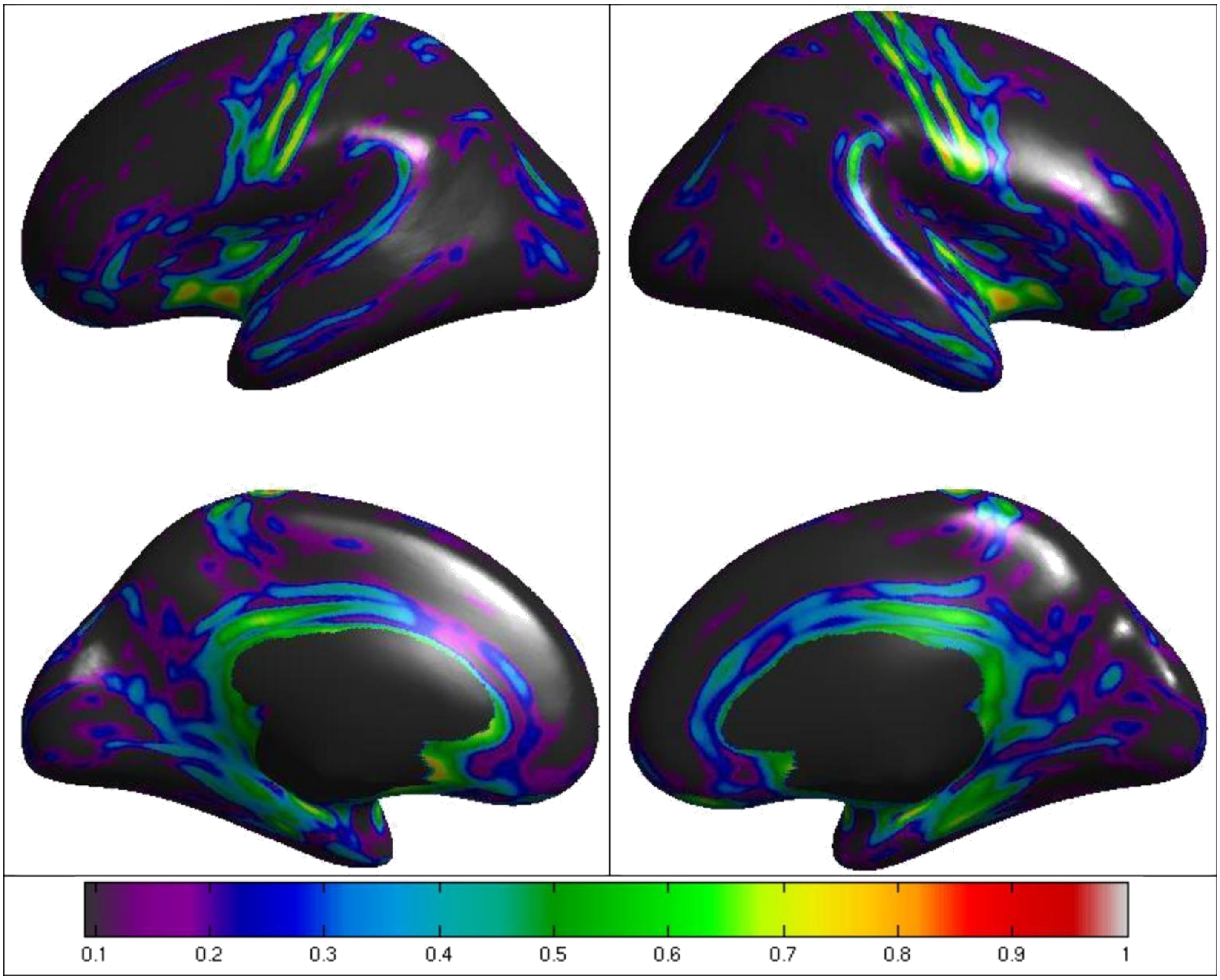

Supplementary Figure S4: Local similarity of cortical thickness profiles maps obtained from a sliding window with a diameter size of 8 mm covering on average 262.8 vertices. Maps were thresholded at $p < 10^{-6}$. Color code expresses average correlation.

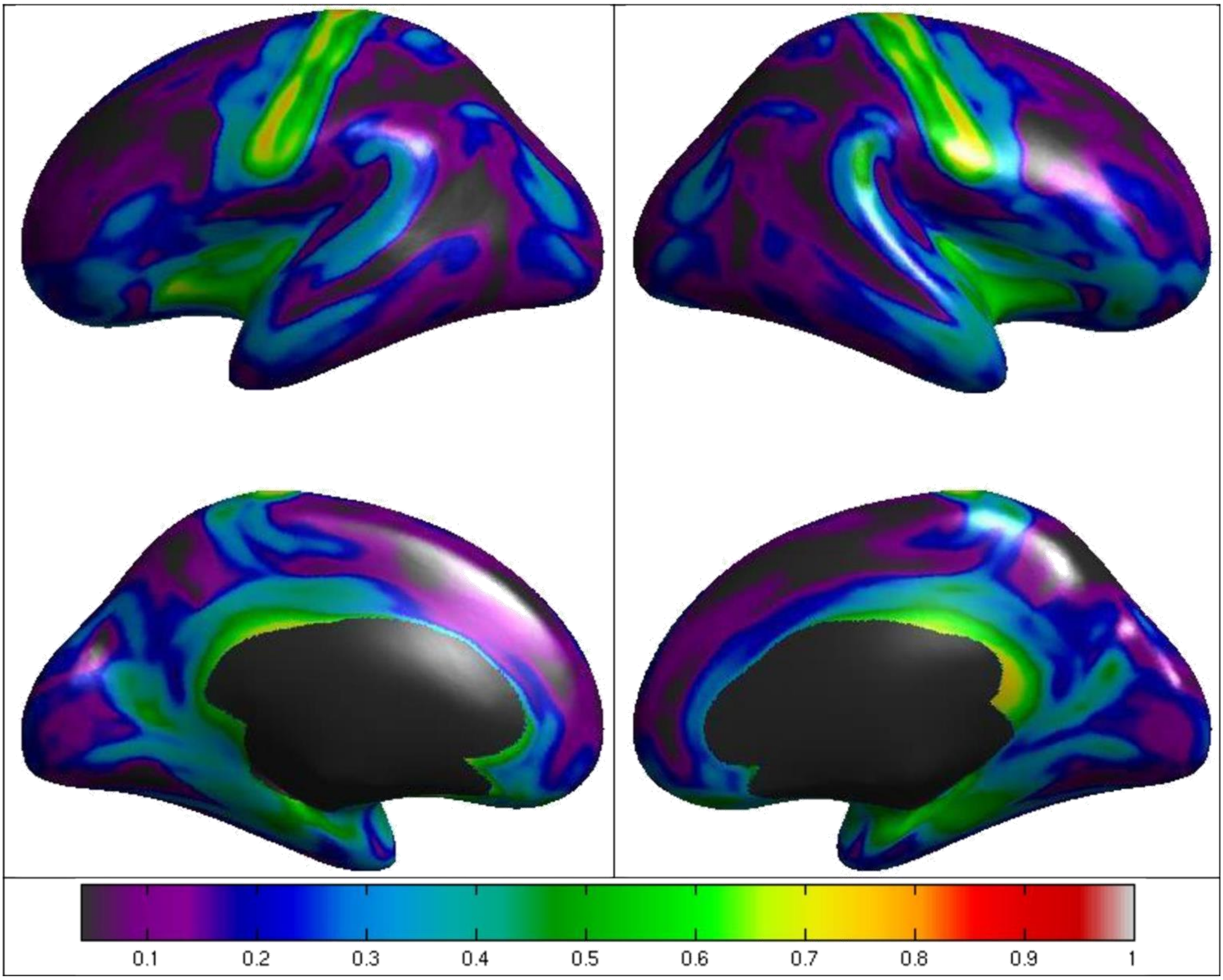

Supplementary Figure S5: LSCTP maps obtained from a sliding window with a diameter size of 16 mm covering on average 1054.3 vertices. Maps were thresholded at $p < 10^{-6}$. Color code expresses average correlation.

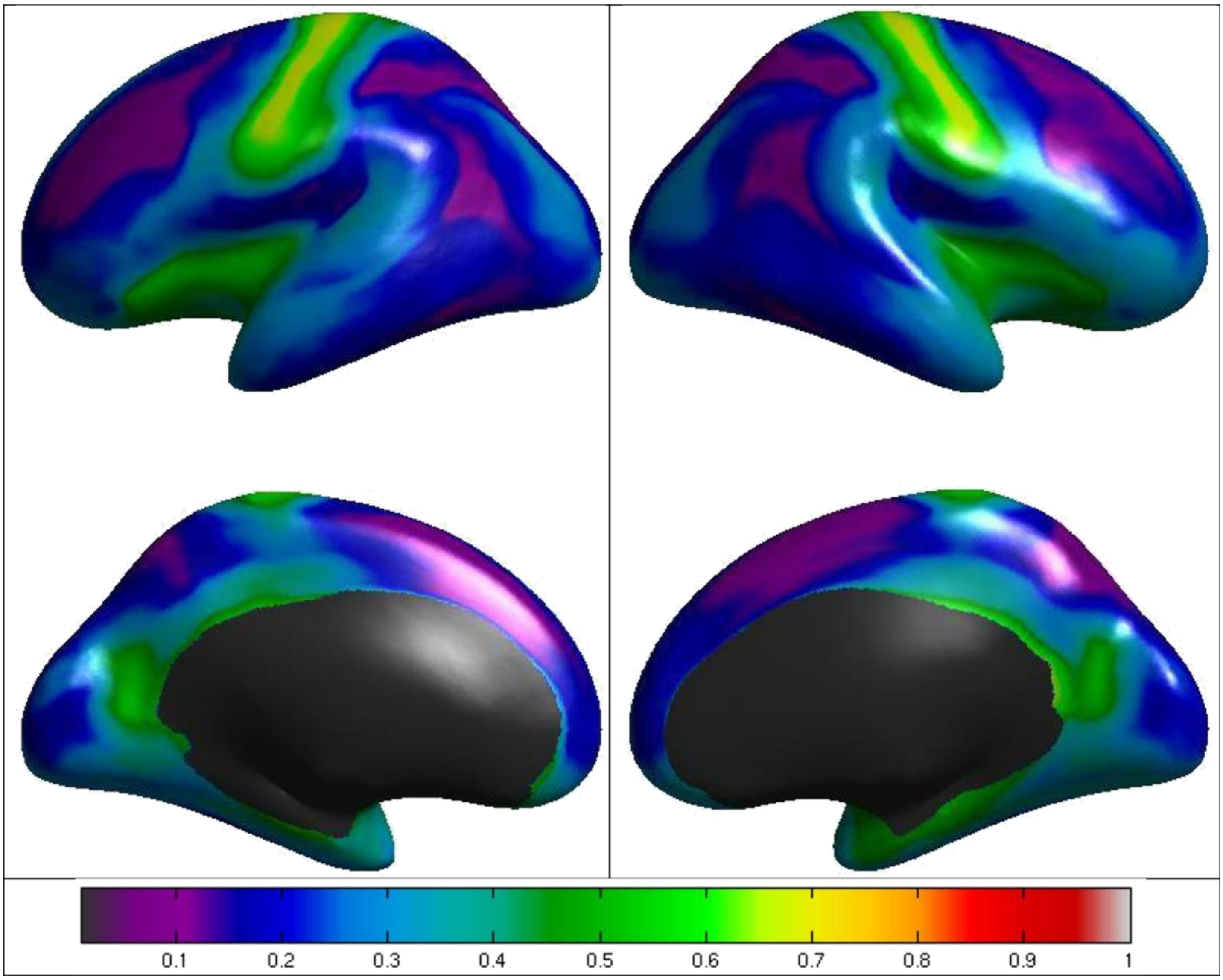

Supplementary Figure S6: LSCTP maps obtained from a sliding window with a diameter size of 32 mm covering on average 4201.4 vertices. Maps were thresholded at $p < 10^{-6}$. Color code expresses average correlation.

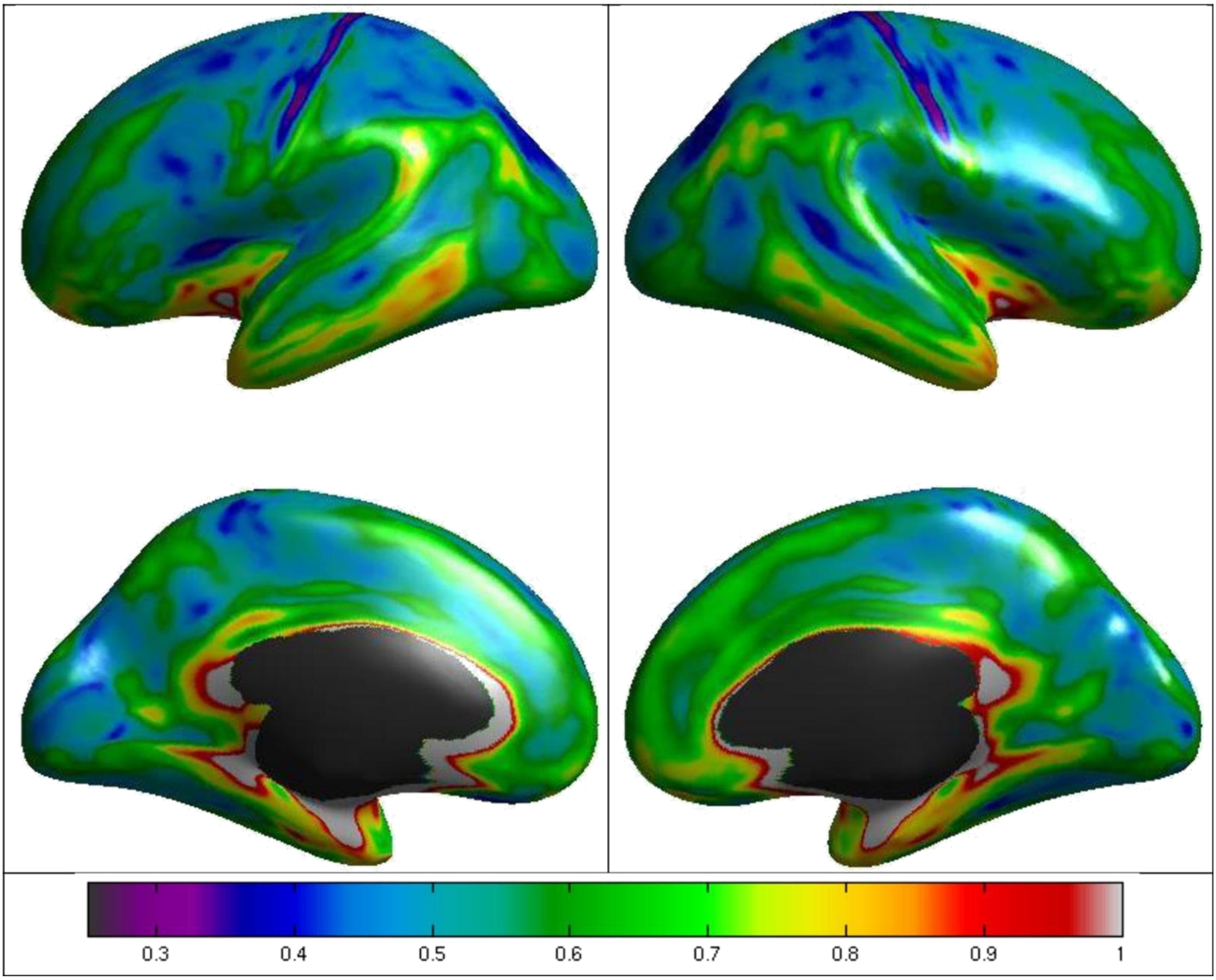

Supplementary Figure S7: Local deviation of cortical thickness maps obtained from a sliding window with a diameter size of 8 mm covering on average 262.8 vertices. Color code expresses standard deviation.

Supplementary Information 3: Monte Carlo simulations and other unlikely events

Here we report the technical details of the Monte Carlo analysis. Supplementary Table S1 reports spatial smoothness of true cortical thickness maps per subject using FS_average smoothwm and FS_average sphere.reg meshes as targets. We show that estimated FWHM indeed differs across different meshes but the number of smoothing iterations needed to obtain shuffled data with the same degree of smoothness as true data is identical. In Supplementary Table S2 and S3 we give the results of the Monte Carlo simulation. Supplementary Table S2 reports results for shuffled data that were smoothed. Table S2 also reports the results of the simulations as obtained from individual and common masks. Table S3 reports the results of the simulations for shuffled data that were not smoothed. Finally, we report the average LSCTP per functional mask per window in Supplementary Table S4.

**observed fwhm FS_average sphere.reg (dv 0.9438)**	**No of iterations**	**observed fwhm FS_average smoothwm (dv 0.6963)**	**No of iterations**
4.92	12	3.63	12
4.72	11	3.48	11
4.77	11	3.52	11
5.42	14	4.00	15
4.98	12	3.68	12
5.15	13	3.80	13
5.18	13	3.82	13
5.21	13	3.85	13
4.64	11	3.42	11
5.06	13	3.73	13
4.92	12	3.63	12
5.13	13	3.78	13
4.70	11	3.47	11
5.08	13	3.75	13
4.98	12	3.67	12
5.02	13	3.70	12
4.73	11	3.49	11
4.75	11	3.50	11
4.86	12	3.59	11
5.10	13	3.76	13
5.43	14	4.01	14
4.87	12	3.60	12
5.57	15	4.11	15
4.76	11	3.51	11
4.54	10	3.35	10
5.08	13	3.75	13
4.84	12	3.57	12
4.63	11	3.42	11
5.27	14	3.89	14
5.03	12	3.71	12
4.80	11	3.54	11
4.80	12	3.54	11
5.05	12	3.72	13
4.47	10	3.30	10
4.94	12	3.64	12
4.58	11	3.38	11
5.01	12	3.70	13
4.96	12	3.66	12
4.78	11	3.52	11
4.87	12	3.59	12
5.10	13	3.76	13
4.71	11	3.48	11
Mean 4.94	Mean 12.0714286	Mean 3.64	Mean 12.0714286

Supplementary Table S1 reports the number of smoothing iterations performed on spatially shuffled data. We also report the spatial smoothness of the observed “true” data that were estimated using the formula (1) found in the main text. “dv” refers to the inter neighbor distance of the respective meshes.

Critical LSCTP threshold for a given sliding window size with smoothing

Individual mask

	sliding window diameter 4 mm	8 mm	16 mm	32 mm	likelihood
Critical LSCTP threshold	0.1039	0.0530	0.0230	0.0108	0.001
	0.1407	0.0700	0.0306	0.0122	0.0001
	0.1857	0.0841	0.0380	0.0131	0.00001
	0.2399	0.0914	0.0412	0.0139	0.000001
mean number of vertex in sliding window	64.4	262.8	1054.3	4201.4	
number of Monte Carlo simulations	5261680	5096380	4776480	4142820	
number of vertices in mesh	327696	327696	327696	327696	
number of vertices in mask	64612	72877	88872	120555	

common mask

	sliding window diameter 4 mm	8 mm	16 mm	32 mm	likelihood
Critical LSCTP threshold	0.1037	0.0531	0.0230	0.0108	0.001
	0.1416	0.0708	0.0307	0.0122	0.0001
	0.1898	0.0846	0.0385	0.0131	0.00001
	0.2476	0.0917	0.0415	0.0139	0.000001
mean number of vertex in sliding window	64.4	262.8	1054.3	4201.4	
number of Monte Carlo simulations	4142820	4142820	4142820	4142820	
number of vertices in mesh	327696	327696	327696	327696	
number of vertices in mask	120555	120555	120555	120555	

Supplementary Table S2: This table reports the results of the Monte Carlo simulations. Critical LSCTP thresholds for every sliding window size are reported. Maps were spatially shuffled on the single subject level. Subsequently shuffled maps underwent smoothing until they approached smoothness of true data. Next LSCTP maps were estimated from shuffled and smoothed maps. The number of smoothing iterations is given in table S1. Results presented on top are based on the number of vertices feasible for a given window size while results presented at the bottom were estimated from vertices within a common mask that was constructed for the LSCTP maps that were investigated with a sliding window size of 32 mm.

Critical LSCTP threshold for a given sliding window size without smoothing

	sliding window diameter 4 mm	8 mm	16 mm	32 mm	likelihood
Critical LSCTP threshold	0.0157	0.0071	0.0033	0.0016	0.001
	0.0196	0.0085	0.0040	0.0019	0.0001
	0.0234	0.0098	0.0047	0.0022	0.00001
	0.0256	0.0106	0.0051	0.0023	0.000001
number of Monte Carlo simulations	4142820	4142820	4142820	4142820	

Supplementary Table S3: This table reports the results of the Monte Carlo simulations. Critical LSCTP threshold for every sliding window are reported when shuffled data were not smoothed.

Average height of LSCTP per functional network and window

window diameter	4	8	16	32
	Mean correlation			
Visual	-0.0002	0.0000	-0.0000	0.0014
Somato Motor	0.0002	0.0002	-0.0002	-0.0004
Dorsal Attention	-0.0002	0.0000	0.0006	0.0001
Ventral Attention	-0.0001	-0.0001	-0.0004	-0.0003
Limbic	0.0005	0.0008	-0.0001	0.0005
Frontoparietal	-0.0005	0.0001	-0.0002	-0.0001
Default	-0.0001	0.0002	-0.0003	0.0003

Supplementary Table S4: reports average height of LSCTP per functional network and window diameter. Maps were spatially shuffled on the single subject level. Subsequently shuffled maps underwent smoothing until they approached smoothness of true data. Next LSCTP values were estimated from shuffled and smoothed data. Subsequently LSCTP values were extracted per functional network and averaged.

Supplementary Information 4: Additional maps

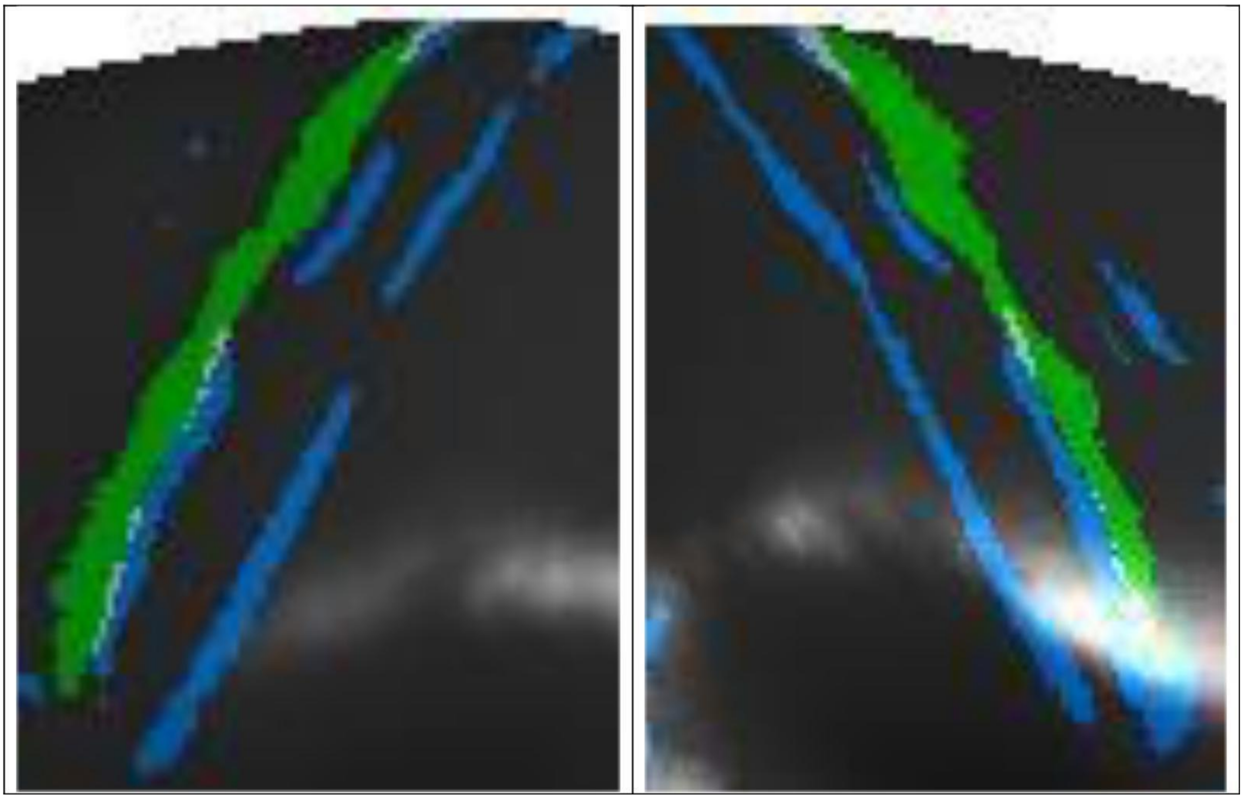

Supplementary Figure S8: LSCTP values > 0.5 that overlap with BA4p are depicted in grey while non-overlapping LSCTP values > 0.5 are depicted in blue

Literature

1). Fischl, B. & Dale, A.M. Measuring the thickness of the human cerebral cortex from magnetic resonance images. *Proc. Natl. Acad. Sci. USA* **97**, 11050–11055 (2000).
